# Corticotropin-Releasing Factor-Producing Cells in the Paraventricular Nucleus of the Hypothalamus and Extended Amygdala Show Age-Dependent FOS and FOSB/DeltaFOSB Immunoreactivity in Acute and Chronic Stress Models in the Rat

**DOI:** 10.3389/fnagi.2019.00274

**Published:** 2019-10-09

**Authors:** László Á. Kovács, Gergely Berta, Valér Csernus, Balázs Ujvári, Nóra Füredi, Balázs Gaszner

**Affiliations:** ^1^Department of Anatomy, University of Pécs Medical School, Pécs, Hungary; ^2^Centre for Neuroscience, Pécs University, Pécs, Hungary; ^3^Department of Medical Biology and Central Electron Microscope Laboratory, University of Pécs Medical School, Pécs, Hungary

**Keywords:** chronic variable mild stress, acute restraint stress, central amygdala, bed nucleus of stria terminalis, PVN

## Abstract

Corticotropin-releasing factor (CRF) immunoreactive (ir) neurons of the paraventricular nucleus of the hypothalamus (PVN) play pivotal role in the coordination of stress response. CRF-producing cells in the central nucleus of amygdala (CeA) and oval division of the bed nucleus of stria terminalis (BNSTov) are also involved in stress adaptation and mood control. Immediate early gene products, subunits of the transcription factor activator protein 1 (AP1) are commonly used as acute (FOS) and/or chronic (FOSB/deltaFOSB) markers for the neuronal activity in stress research. It is well known that the course of aging affects stress adaptation, but little is known about the aging-related stress sensitivity of CRF neurons. To the best of our knowledge, the stress-induced neuronal activity of CRF neurons in the course of aging in acute and chronic stress models was not studied systematically yet. Therefore, the aim of the present study was to quantify the acute restraint stress (ARS) and chronic variable mild stress (CVMS) evoked neuronal activity in CRF cells of the PVN, CeA, and BNSTov using triple-label immunofluorescence throughout the whole lifespan in the rat. We hypothesized that the FOS and FOSB content of CRF cells upon ARS or CVMS decreases with age. Our results showed that the FOS and FOSB response to ARS declined with age in the PVN-CRF cells. BNSTov and CeA CRF cells did not show remarkable stress-induced elevation of these markers neither in ARS, nor in CVMS. Exposure to CVMS resulted in an age-independent significant increase of FOSB/delta FOSB immunosignal in PVN-CRF neurons. Unexpectedly, we detected a remarkable stress-independent FOSB/deltaFOSB signal in CeA- and BNSTov-CRF cells that declined with the course of aging. In summary, PVN-CRF cells show decreasing acute stress sensitivity (i.e., FOS and FOSB immunoreactivity) with the course of aging, while their (FOSB/deltaFOSB) responsivity to chronic challenge is maintained till senescence. Stress exposure does not affect the occurrence of the examined *Fos* gene products in CeA- and BNSTov-CRF cells remarkably suggesting that their contribution to stress adaptation response does not require AP1-controlled transcriptional changes.

## Introduction

Neuronal activity in functional–morphological studies is frequently assessed by quantitation of immediate early gene (IEG) products. For instance, FOS (Sagar et al., 1988) and FOSB (Chen et al., 1997) are widely used tools to test if neurons react by changes at transcriptional level to various stimuli (for reviews see Kovács, 1998, 2008). These proteins are products of the *Fos* proto-oncogene family and contribute to the modulation of gene expression as subunits of the well-known transcription factor activator protein 1 (AP1). *Fos* is a commonly used neuronal activity marker to study the acute neuronal response that occurs minutes after the stimulus at mRNA level (Sawchenko et al., 1996). At protein level, FOS peaks 2 h after the stimulus (Kovács, 2008) and finally returns to baseline in 4–6 h (Sonnenberg et al., 1989).

Another member of this protein family is FOSB. *FosB* shows increase both in its transcription and translation to FOSB protein upon exposure to stimuli that require neuronal response at the level of gene expression (Sonnenberg et al., 1989). FOSB exerts slower dynamics than FOS (Morgan and Curran, 1989) with its half-life of 9.5 h (for reviews see Kovács, 1998, 2008). Importantly, a splice variant of FOSB protein designated as deltaFOSB (ΔFOSB) was shown to exert an even more prolonged dynamics. Multiple exposures to the stimuli are required to increase its level that stays high for a longer period of time (i.e., days). Therefore, ΔFOSB is a useful tool to visualize chronic neuronal activity (Nestler et al., 1999; Perrotti et al., 2004).

Several factors are known to influence the occurrence of these activity markers (for review see Kovács, 2008). The exposure to stressful stimuli was also repeatedly shown to affect the magnitude of neuronal stress response that is quantified by the assessment of FOS and/or FOSB immunoreactivities.

Sporadic literature data suggested that stress sensitivity in terms of FOS immunoreactivity (ir) might be affected by age (Kellogg et al., 1998; Viau et al., 2005; Romeo et al., 2006; Meyza et al., 2007). In our previous study (Kovács et al., 2018), we assessed the FOS sensitivity of numerous stress centers in the rat brain throughout the whole lifespan systematically. We showed that the magnitude of FOS rise elicited by acute stress exposure was also a function of age in stress adaptation centers of the rat. In that study we found that besides several other brain areas, the paraventricular nucleus of the hypothalamus (PVN), the central nucleus of the amygdala (CeA), and the oval division (BNSTov) of the bed nucleus of the stria terminalis (BNST) also showed age dependency. As these brain areas are major corticotropin-releasing factor (CRF)-producing sites in the brain (Swanson et al., 1983; Merchenthaler, 1984; Sawchenko and Swanson, 1985), the question arose, if the age-dependent FOS response to stress is specific for the CRF neurons.

Corticotropin-releasing factor produced by the parvocellular division of the PVN is the main regulator of the stress response via the hypothalamus–pituitary–adrenal (HPA) axis. Indeed, PVN *Crf* expression increases upon stress exposure at mRNA level (Imaki et al., 1995; Choi et al., 2008; Sterrenburg et al., 2012) in the rat. It is well-documented that acute (Gaszner et al., 2012; Sterrenburg et al., 2012; Kovács et al., 2018) and chronic stress exposure (Sterrenburg et al., 2011) results in the rise of FOS and FOSB immunoreactivities in rodent PVN neurons, respectively (for reviews see Kovács, 1998, 2008). Importantly, the *Crf* gene promoter contains AP-1 binding sites which control the expression of *Crf* mRNA in interaction with glucocorticoids (Malkoski and Dorin, 1999). After translation and axonal transport to the median eminence, the CRF peptide is released into the portal circulation of the anterior lobe of the pituitary to stimulate the corticotroph cells. The latter cells secrete adrenocorticotropin that controls the glucocorticoid hormone release in the *zona fasciculata* of the adrenal cortex. In rodents, corticosterone (CORT) controls a wide spectrum of physiological functions affecting the stress response (Herman and Cullinan, 1997; Deussing and Chen, 2018) immune system (Walker and Spencer, 2018), energy metabolism (de Kloet and Herman, 2018), reproduction and growth (Joseph and Whirledge, 2017).

Limbic areas, such as nuclei of the extended amygdala, were also shown to contribute to the stress adaptation response (for review see Ulrich-Lai and Herman, 2009). For instance, the medial and central nuclei of the amygdala facilitate the stress response of the HPA axis indirectly by suspending the GABAergic tonic inhibition of the peri-PVN area on the CRF neurons (for review see Herman et al., 2016). Interestingly, the CeA harbors a larger population of cells which show CRF ir in the rat (Swanson et al., 1983; Merchenthaler, 1984). CeA-CRF neurons project to important centers of the stress response, such as the BNST, periaqueductal gray matter, and locus ceruleus (LC) (Dong et al., 2001). Local overexpression of CRF induced by a lentiviral vector was found to dampen stress-induced anxiety in mice (Regev et al., 2012). More recently, the local CRF circuit was shown to be involved in discriminative fear in mice (Sanford et al., 2017). In contrast to findings in mice, in the rat, the overexpression of CRF in the CeA resulted in increased anxiety. Interestingly, the phenotype effect of CRF overexpression occurred in female rats after the onset of their puberty (Flandreau et al., 2012), and the overexpression was shown to bring forward puberty in time (Li et al., 2014), suggesting the significance of CeA-CRF cells in this period of life. The existence of IEG products was also shown in the CeA both in acute (Romeo et al., 2006; Sterrenburg et al., 2012) and chronic stress models (Sterrenburg et al., 2011) in the rat. In our previous study we found that the magnitude of FOS response to acute restraint stress (ARS) was age dependent in the rat CeA (Kovács et al., 2018). The question, if the age-related decrease of FOS ir is characteristic for the CRF-producing cells in the CeA remained to be answered.

Another important CRF-producing site of the extended amygdala is the dorsal division of the BNST (Merchenthaler, 1984; Sawchenko and Swanson, 1985; Kozicz et al., 1997). According to the classification of Dong et al. (2001), the oval division contains CRF immunoreactive (ir) cells which project to the PVN and contribute to the control of mood status also. For instance, lentiviral overexpression of CRF in these cells induces depression-like phenotype in mice (Regev et al., 2012). Acute stress results in FOS production in the BNSTov in mice (Gaszner et al., 2012) and rats (Sterrenburg et al., 2012). Exposure to chronic stress causes increased FOSB immunoractivity in BNSTov neurons in mice (Kormos et al., 2016) and rats (Sterrenburg et al., 2011), however, if mice were previously exposed to early life stress also, BNSTov-CRF neurons fail to react to the chronic stress exposure (Farkas et al., 2017). In our most recent work (Kovács et al., 2018) we found that the magnitude of BNSTov FOS ir upon ARS exposure declined with the course of aging, but if this alteration would affect the CRF ir neurons was not declared.

If the age-related decline of IEG product content is specific for FOS in acute stress, or, it is characteristic for FOSB also, and if a similar aging-associated decrease is found upon chronic stress exposure was also to be studied. Therefore, we put forward the hypotheses that both (a) acute and (b) chronic stress reactivity of CRF neurons in the PVN, CeA, and BNSTov was affected by the course of aging in the rat. To test these hypotheses, (a) male rats of eight age groups [i.e., 1-month-old (1M), 1.5M, 2M, 3M, 6M, 12M, 18M, and 24M) were exposed to ARS vs. controls. In order to test the effect of aging on responsivity of CRF neurons to chronic stress, (b) six groups of rats (2M, 3M, 6M, 12M, 18M, and 24M) were exposed to chronic variable mild stress (CVMS). Using semi-quantitative triple labeling immunofluorescence, CRF, FOS, and FOSB immunoreactivities were studied in the PVN, CeA, and BNSTov. The efficacy of stress exposure was assessed by physical parameters, forced swim test (FST), and plasma CORT measurements.

## Materials and Methods

### Animals

One hundred and fifty-seven albino male Wistar rats bred in the animal facility of the Department of Anatomy (University of Pécs) were used. Eight age groups were formed: 1M, 1.5M, 2M, 3M, 6M, 12M, 18M, and 24M. Animals were housed in two or three rats per standard polycarbonate cage (40 × 25 × 20 cm) on neutral temperature (24°C) in humidity controlled environment. The rooms of the facility were illuminated for 12 h per day with a light phase starting at 6:00 am. Rats were provided with standard rodent chow and tap water *ad libitum*. Animals were handled three times per week when the regular cage cleaning was also performed. Once a week, the animals’ bodyweight was also registered. The studies were approved by the Ethics Committee on Animal Research of Pécs University (license No: BA02/2000-25/2011) based on the European Communities Council Directive of 24 November 1986 and the Law of 1998, XXVIII, on Animal Care and Use in Hungary. All efforts were made to minimize the number of animals used and their suffering.

### Acute Restraint Stress Protocol

Acute restraint stress exposure was performed exactly, as previously published (for details see Kovács et al., 2018) in all eight age groups (*n* = 4–7). Briefly, rats were closed into custom-made polycarbonate restrainer tubes with several ventilation holes. For 1M rats a 30 mm, for 1.5M a 35 mm, and for 2M old animals 40 mm diameter restrainer tubes were used. For all older groups a diameter of 45 mm was preferred. After 60 min restraint, rats were returned to their original home cages for 60 min as the maximum of FOS ir was expected 2 h after the start of stress exposure (Kovács, 2008). Control rats of all age groups were left undisturbed in their home cages.

### Chronic Variable Mild Stress Protocol

The CVMS protocol was not adaptable to the pre-weaning period, therefore the effect of CVMS was tested only in six age groups (i.e., 2M, 3M, 6M, 12M, 18M, and 24M) with *n* = 6–8 rats. The 14 days long stress protocol consisted of a shorter stress session during the light phase of the day and a longer night-time stress exposure. The daytime stress was applied between 10 am and 2 pm as one of the following challenges: shaker (animals with their cages were placed on an orbital laboratory shaker with frequency of 80 rpm for 120 min), tilted cage (the home cage of animals was fixed in a 45° tilted position for 180 min), dark room (cages were placed in a completely dark room of the animal facility for 180 min), and restraint (60 min of ARS, as described above). The night-time stress exposure started at 6:00 pm and finished 6:00 am in the next morning. For night-time stress exposure either social isolation (animals were separated individually in cages with fresh nesting material) or wet bedding (the woodchips was moisturized with 400 ml tap water overnight followed by cage cleaning and nesting material change at 6:00 am). These two stressors were randomly applied, and approximately every third night, groups of rats were left undisturbed in their cages (group holding).

In order to test the behavioral effect of CVMS exposure, an independent cohort of CVMS-exposed 6M (*n* = 12) animals with age matched controls (*n* = 12) was subjected to FST according to Porsolt et al. (1977). In these rats, on day 14 of CVMS protocol, 15 min FST was performed (pre-test day) using a glass cylinder (diameter 25 cm, height 40 cm) filled with 24°C water till 30 cm. One day later, a 5 min FST was applied and the cumulative immobility time of rats was measured on video recordings. Animals used for behavioral testing were not included into the morphological assessment.

### Tissue Collection and Sample Preparation

Control animals were deeply anesthetized in their home cages by an overdose of intraperitoneal urethane (2.4 g/kg) injection between 10 and 11 am. ARS rats were injected the same way 60 min after the end of restraint stress exposure. Rats of CVMS groups were euthanized on day 15, 24 h after the start of the last daytime restraint stress. In order to avoid the acute stress effect of anesthetic injection on CORT values, only those rats were used in this experiment which got unconscious within 2 min after injection.

After successful anesthesia, the chest cavity was quickly opened, and a small cut was made on the left ventricle and a blood sample for CORT measurement (see later) was collected. Then, rats were transcardially perfused with 50 ml 0.1 M phosphate-buffered saline (PBS, pH = 7.4) followed by 250 ml ice cold 4% formaldehyde solution in 0.2 M Millonig sodium-phosphate buffer (pH = 7.4) in 20 min. Finally, animals were decapitated and their brains were dissected and post-fixed in the same fixative for 72 h. Thymus and adrenals were also collected and weighed.

Thirty-micrometer coronal sections were cut between the optic chiasm and ponto-medullary transition using Leica Vibratome (Leica Biosystems, Wetzlar, Germany). Three series of sections each interspaced by 90 μm were collected into anti-freeze solution (30% glycerol, 20% ethylene-glycol, 0.1 M PBS) and stored on −20°C till further examination. Before starting the labeling, sections containing the PVN [(−1.56 mm) to (−1.92 mm) to the Bregma], CeA [(−2.40 mm) to (−2.92 mm) to the Bregma], and BNST [+0.12 mm to (−0.24 mm) to the Bregma] were manually selected according to the rat brain atlas by Paxinos and Watson (2007).

### Free-Floating Triple Immunofluorescence for FOS, FOSB, and CRF

Selected sections were rinsed 4 × 10 min in PBS. A heat-induced epitope retrieval was applied at 90°C in citrate buffer (pH = 6.0) for 10 min. Sections were left in the same solution to cool down to room temperature for additional 20 min. Then, sections were washed for 2 × 10 min in PBS and permeabilized with 0.5% Triton X-100 for 60 min (Sigma–Aldrich Kft, Budapest, Hungary). Subsequently, after an incubation for 60 min in 2% normal donkey serum (NDS, Jackson Immunoresearch Europe Ltd., Suffolk, United Kingdom) in PBS, sections were treated with the mixture of polyclonal rabbit CRF antiserum diluted to 1:16,000 (gift from Prof. WW Vale, The Salk Institute, La Jolla, CA, United States), FOS polyclonal guinea pig antiserum diluted to 1:1000 (Synaptic System GmbH, Goettingen, Germany, Cat. No.: 226004), and polyclonal mouse FOSB antiserum diluted to 1:1500 (Abcam, Cambridge, United Kingdom; AB11959) in NDS for 48 h at 4°C. After 2 × 15 min PBS washes, sections were incubated for 24 h in a cocktail of the following secondary antisera purchased from Jackson: biotinylated donkey anti-rabbit IgG (1:500), Alexa 488-conjugated donkey anti-guinea pig IgG (1:600), Cy3-conjugated donkey anti-mouse IgG diluted to 1:400 in PBS. After repeated washes in PBS, preparations were treated with Cy5 streptavidine (1:1000, Jackson) for 3 h. To avoid the influence of the autofluorescence due to aging-related lipofuscin accumulation, an autofluorescence eliminator kit was used according to the supplier’s instructions with slight modifications (Merck KgaA, Darmstadt, Germany, Cat. No.: 2160, Lot No.: 2016635). Sections after immersion in PBS for 5 min were transferred into 30% and then into 70% ethanol solution for 5 min, respectively. Then, sections were immersed into the autofluorescence eliminator reagent for 2 min, followed by 2 × 1 min treatment with 70% ethanol solution. Finally, sections were rinsed 2 × 15 min in PBS, mounted on gelatin-covered slides, dried on air, and covered with a solution of PBS and glycerol (1:1).

The specificity and sensitivity of the FOS antiserum (Synaptic Systems GmbH, Goettingen, Germany, Cat. No.: 226004) was tested on our samples. Omission of the primary or secondary antiserum, their replacement with normal non-immune sera abolished the immunosignal in this experiment. Pre-incubation of the working dilution of the antiserum with the respective blocking peptide (Synaptic Systems, Cat. No.: 226-0P) prevented the immunolabeling also (Supplementary Figure 1). The FOSB serum was pre-tested (Supplementary Figure 1) and used based on the producer’s suggestions. Earlier works (Tuplin et al., 2018) as well as Western blot analysis as published at the supplier’s webpage support the specificity of this serum. The CRF serum produced in rabbit (PBL# rC70) generously provided by Prof. WW Vale’s laboratory is a widely used well-trusted serum with high specificity and sensitivity (Supplementary Figure 1). During the pre-tests, the sensitivity of this antibody was compared with that of a previously commercially available also reliable goat CRF serum (Santa Cruz, see also Kormos et al., 2016). Both sera gave a clear co-localizing cytoplasmic immunosignal in BNSTov and CeA. However, as the rabbit CRF serum provided a stronger signal in the PVN, this antibody was preferred. For further details on antibodies the reader is referred to Supplementary Table 1.

### Microscopy, Digitalization, and Morphometry

An experienced neuro-histologist colleague who was unaware of the identity of the preparations digitalized the sections containing the PVN, CeA, and BNST areas by Olympus FluoView 1000 confocal microscope. Sequential scanning in photon count mode was used for the respective fluorophores to detect semi-quantitative fluorescent signal. The confocal aperture was set to 80 μm. The scanning was performed with a 20× objective to obtain images of 1024 × 1024 pixel resolution. The excitation and emission spectra of the respective fluorophores were set according to the built-in settings of the Fluo-View software (Fv10-ASW; Version 0102). 488, 550, and 633 nm laser beams were used to excite Alexa 488 (emission peak for detection: 525 nm), Cy3 (emission peak for detection: 570 nm), and Cy5 (emission peak for detection: 670 nm), respectively.

Images of respective channels were stored both individually, and superimposed to evaluate the co-localization of fluorescent signals. Red (Cy3), green (Alexa 488), and white (Cy5) virtual colors were used.

The number of the cells was counted in non-edited digital images by a colleague who wasn’t informed about the identity of sections and the purpose of the study. The quantitation of neurons was performed on five digital images of each brain area of interest selected from one series of representative sections. All cells were counted on the entire cross-section surface of the respective nuclei. Cell counts of the five sections were averaged. These numbers represented one animal in the statistical analysis. In order to provide a semi-quantitative analysis of neuronal CRF content, the intensity of fluorescence in the perikarya was measured and corrected for the background signal, yielding the specific signal density (SSD, for further details see Kormos et al., 2016) using Image J software (version 1.50i, NIH). The measurement was performed on manually selected CRF cells which were positive for (a) FOS, (b) FOSB, and (c) for both FOS and FOSB. Finally, the CRF SSD was also measured in cells which did not contain these activity markers and the SSD of CRF was correlated with the other registered variables.

For publication purposes, selected representative digital images were contrasted, cropped, and edited into montages by Adobe Photoshop 7.0.1 software.

### Corticosterone Radioimmunoassay

The determination of blood CORT levels was performed as published earlier (Gaszner et al., 2004, 2009). Briefly, 1 ml left ventricular blood was collected right before perfusion into pre-chilled plastic tubes containing 150 μl 8 m/m% EDTA solution. After centrifuge at 3500 rpm for 5 min at 4°C the plasma fraction was isolated and 50 μl samples were stored at −80°C till further analysis.

The dried extract of 5 μl plasma was reconstituted with assay buffer. From each sample two independent measurements were performed. The vials contained 500 μl extract, tritiated CORT (12,000 cpm; NET-399, 90–120 Ci/mmol Perkin Elmer, Akron, OH, United States) and 15 nl/tube CS-RCS-57 antibody (1:47,000; Jozsa et al., 2005) in 700 μl total volume and were incubated for 16 h at 4°C. Then, bound and free steroids were separated with dextran-coated charcoal and the radioactivity was measured in a two-phase liquid scintillation system. Calbiochem CORT was used as a standard. The inter- and intra-assay coefficients for variation were 9.03% and 4.5%, respectively.

### Statistical Analysis

Data were presented as mean of groups ± standard error of the mean (SEM). The normality of data distribution and the homogeneity of variance were verified by Shapiro–Wilk (Shapiro and Wilk, 1965) and Hartley’s Chi-square tests (Snedecor and Cochran, 1989), respectively. To obtain normal distribution, some data were subjected to logarithmic transformation. Statistical analyses were performed by two-way analysis of variance (ANOVA) followed by Tukey’s *post hoc* tests. To further assess the relationship between datasets, the Spearman’s rank correlation test was used. FST results of CVMS exposed rats vs. controls were assessed by Student’s *t*-test. The statistical difference was considered significant if alpha was <5%. The statistical analyses were conducted using Statistica 8.0 software (StatSoft, Tulsa, OK, United States).

## Results

### Presence of IEG Products in CRF Neurons Upon Acute Restraint Stress

In order to test the potential age dependency in CRF neuronal activity upon acute stress exposure, the FOS and FOSB immunoreactivities were assessed in the PVN, CeA, and BNSTov. Eight age groups of rats were compared with or without ARS exposure.

#### FOS in the CRF Immunoreactive Neurons of the Paraventricular Nucleus

**FIGURE 1:**
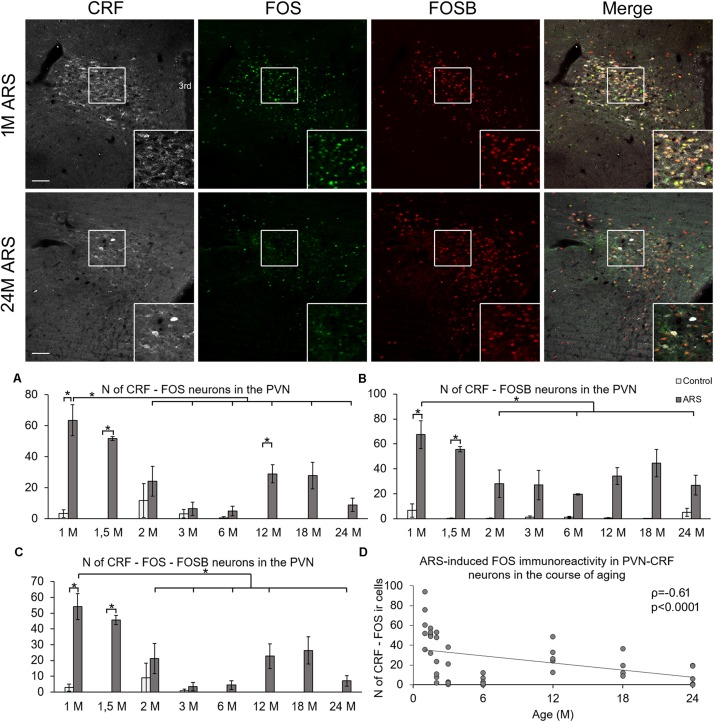
Age-dependent FOS and FOSB immunoreactivities in the paraventricular nucleus of the hypothalamus (PVN). Representative images depicting the PVN area of 1-month-old (1M) and 24M acute restraint stress (ARS) exposed rats. Note that most of the CRF (white) immunoreactive (ir) cells contain both FOS (green) and FOSB (red) as also shown by the merged image on the right in 1M animals. The immunosignal for both FOS and FOSB declined with age as represented by images of 24M rats. Histogram **(A)** shows the number (N) of CRF and FOS-containing cells in control (open bars) and ARS exposed (gray columns) rats in eight age groups. Panel **(B)** demonstrates the N of cells where CRF and FOSB co-exist. Chart **(C)** demonstrates the aging-related change in the number of CRF cells which contain both FOS and FOSB. Significant differences according to the Tukey’s *post hoc* tests were highlighted by asterisks (*p* < 0.05, *n* = 4–8). **(D)** A scatter plot of the Spearman’s correlation test illustrates the age-related decline of the N of CRF-FOS labeled cells in ARS. Boxed areas are depicted in higher magnification insets in the right bottom corner of the respective images. 3rd, third ventricle. Scale bar 100 μm.

Analysis of variance (Table 1) revealed that both age (*F*_(__7__,__78__)_ = 74.39; *p* < 10^–6^) and acute stress (*F*_(__1__,__78__)_ = 7.72; *p* < 10^–5^) affected the number of CRF-FOS double labeled cells (Figure 1A). Moreover, the statistical analysis confirmed the interaction of these factors also (*F*_(__7__,__78__)_ = 7.68; *p* < 10^–5^). Tukey’s *post hoc* test revealed that 1M, 1.5M, and 12M animals upon acute stress exposure showed significantly elevated CRF-FOS cell counts compared to their respective controls (*p* < 0.0005). Spearman’s correlation test (Table 2) verified a moderate negative correlation between PVN’s CRF-FOS cell count and age (ρ = −0.61; *p* < 0.0001, Figure 1D). The ARS animals’ PVN, CRF-FOS cell counts correlated with the CRF-FOSB cell counts (ρ = 0.76; *p* < 10^–6^).

**TABLE 1:** Summary of statistical results obtained by two-way analysis of variance (ANOVA).

**Groups**	**Cells labeled for**	**Area**	**Two-way ANOVA**					
			**Main effects**				**Interaction**	
			**Age**		**Stress**		**Age × stress**	
			***F*^∗^**	***p***	***F*^∗^**	***p***	***F*^∗^**	***p***
Controls vs. ARS	CRF-FOS	PVN	**7.71**	**<10^–5^**	**74.39**	**<10^–6^**	**7.68**	**<10^–5^**
		BNSTov	1.64	0.14	0.21	0.66	1.42	0.21
		CeA	1.26	0.28	0.22	0.64	1.60	0.15
	CRF-FOSB	PVN	**2.61**	**<0.05**	**89.17**	**<10^–6^**	**3.03**	**<0.01**
		BNSTov	**5.32**	**<10^–4^**	**4.13**	**<0.05**	1.18	0.32
		CeA	**3.45**	**<0.005**	**6.04**	**<0.05**	0.74	0.64
	CRF-FOSB-FOS	PVN	**7.35**	**<10^–5^**	**66.81**	**<10^–6^**	**6.78**	**<10^–5^**
Controls vs. CMVS	CRF-FOSB	PVN	2.19	0.07	**64.19**	**<10^–6^**	1.93	0.11
		BNSTov	**5.37**	**<0.001**	0.06	0.81	0.97	0.44
		CeA	**3.93**	**<0.005**	0.91	0.34	0.65	0.66

*The FOS and FOSB expression of corticotropin-releasing factor (CRF) immunoreactive neurons was studied in the paraventricular nucleus of the hypothalamus (PVN), in the oval division of the bed nucleus of the stria terminalis (BNSTov) and in the central nucleus of the amygdala (CeA). Control rats were compared with age matched acute restraint stress (ARS) and chronic variable mild stress (CVMS) exposed counterparts. ^∗^The degrees of freedom for the comparison between control and ARS were *F*_(__1,78__)_ for stress and *F*_(__7,78__)_ for age and for age × stress interaction, respectively. In the control vs. CVMS comparison the following degree of freedom values applied: *F*_(__1,63__)_ for stress and *F*_(__5,63__)_ for age and age × stress interaction, respectively. Significant statistical values were highlighted in bold.*

**TABLE 2:** Summary of statistical results by Spearman’s rank correlation tests in acute restraint stress exposed rats.

**Area**	**Labeling**	**Comparison with**	**ρ**	***p***
PVN	CRF-FOS	Age	−0.61	<0.000
		PVN CRF-FOSB	0.76	<10^–6^
		CeA CRF-FOSB	0.41	<0.05
		BNSTov CRF-FOSB	0.32	
hspace*5pt0.06				
		PVN CRF-FOSB-FOS	0.92	<10^–6^
	CRF-FOSB	Age	−0.48	<0.01
		PVN CRF-FOS	0.76	<10^–6^
		CeA CRF-FOSB	0.21	0.24
		BNSTov CRF-FOSB	0.16	0.36
		PVN CRF-FOSB-FOS	0.86	<10^–6^
	CRF-FOSB-FOS	Age	−0.38	<0.001
		PVN CRF-FOS	0.92	<10^–6^
		PVN CRF-FOSB	0.86	<10^–6^
		CeA CRF-FOSB	0.28	<0.05
		BNSTov CRF-FOSB	0.23	0.06
		CRF SSD in CRF-FOSB-FOS cells	0.73	<0.0001
		CRF SSD in cells negative for IEG products	0.35	0.08
BNSTov	CRF-FOSB	Age	−0.64	<0.0001
		PVN CRF-FOS	0.32	0.06
		PVN CRF-FOSB	0.16	0.36
		CeA CRF-FOSB	0.86	<10^–6^
		PVN CRF-FOSB-FOS	0.23	0.06
CeA	CRF-FOSB	Age	−0.42	<0.05
		PVN CRF-FOS	0.42	<0.05
		PVN CRF-FOSB	0.21	0.24
		BNSTov CRF-FOSB	0.86	<10^–6^
		PVN CRF-FOSB-FOS	0.28	<0.05

*The number of FOS or FOSB immediate early gene (IEG) product containing corticotropin-releasing factor (CRF) immunoreactive neurons of the paraventricular nucleus of the hypothalamus (PVN), the oval division of the bed nucleus of the stria terminalis (BNSTov) and central nucleus of the amygdala (CeA) was correlated with data depicted in the third column in acute restraint stress exposed rats. SSD, specific signal density.*

#### FOSB in the CRF Cells of the Paraventricular Nucleus

Analysis of variance (Table 1) revealed the main effect of stress (*F*_(__1__,__78__)_ = 89.17; *p* < 10^–6^), age (*F*_(__7__,__81__)_ = 2.67; *p* < 0.05), and their interaction (*F*_(__7__,__78__)_ = 3.03; *p* < 0.01) on the number of CRF-FOSB double positive cells significant (Figure 1B). The *post hoc* test confirmed a significant increase in CRF-FOSB cell counts in ARS groups vs. their age-matched controls in 1M and 1.5M groups (*p* < 0.0005). Age negatively correlated (Table 2) with the count of CRF-FOSB co-localizing cells in ARS animals (ρ = −0.48; *p* < 0.0005). The ARS-induced PVN-CRF-FOSB cell count positively correlated with CRF-FOS (ρ = 0.76; *p* < 10^–6^).

#### FOSB and FOS in the CRF Neurons of the Paraventricular Nucleus

Two-way ANOVA (Table 1) denoted the main effect of stress (*F*_(__1__,__78__)_ = 7.35; *p* < 10^–5^), age (*F*_(__7__,__78__)_ = 66.81; *p* < 10^–6^), and their interactions (*F*_(__7__,__78__)_ = 6.78; *p* < 10^–5^) on the number of CRF-FOS-FOSB triple positive cells significant (Figure 1C). *Post hoc* analyses proved a significant cell count rise in 1M (*p* < 0.0005) and 1.5M (*p* < 0.0005) ARS rats. The Spearman’s correlation analysis (Table 2) found a weak negative correlation with age (ρ = −0.38; *p* < 0.001). The analysis of CRF SSD revealed that the CRF content of triple positive cells correlated with age (ρ = 0.43; *p* < 0.03) and with IEG product content (ρ = 0.73; *p* < 0.0001), while the CRF content of cells which were immuno-negative for IEG products did not show significant correlation (ρ = 0.35; *p* = 0.08) with the count of IEG product double positive cells. It should be pointed out also that if one activity marker was present in the nucleus of the CRF neurons upon acute stress, the other mostly co-existed also as both CRF-FOSB (ρ = 0.86; *p* < 10^–6^) and CRF-FOS (ρ = 0.92; *p* < 10^–6^) strongly correlated with CRF-FOSB-FOS (Table 2).

#### CRF Neurons in the Extended Amygdala

**FIGURE 2:**
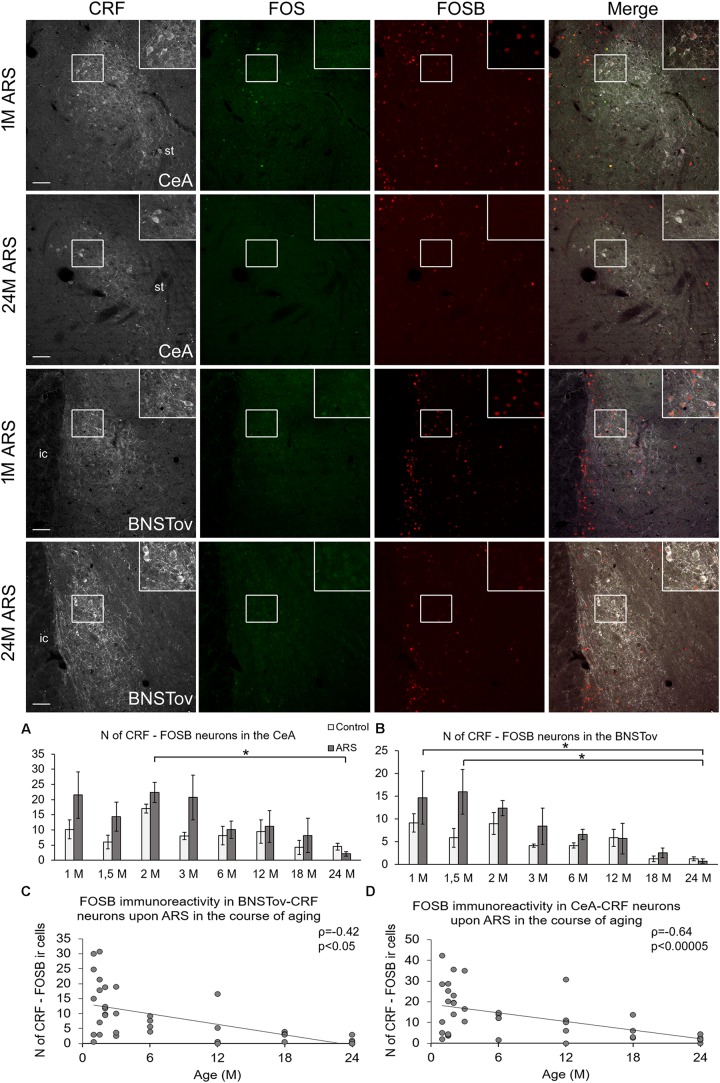
FOSB and FOS immunoreactivities in the central nucleus of the amygdala (CeA) and in the oval division on the bed nucleus of the stria terminalis (BNSTov) upon acute restraint stress (ARS) in 1-month-old (1M) and 24M rats. Representative images of the CeA and BNSTov show that CRF-containing (white) cells do not contain FOS (green) upon ARS. In contrast, CRF cells in 1M animals show FOSB (red) immunoreactivity that declines with age. As shown in histogram **(A)** for the CeA and **(B)** for BNSTov, the FOSB signal does not increase upon ARS exposure (gray bars) in comparison to age-matched controls (open columns). Significant differences according to the Tukey’s *post hoc* tests were highlighted by asterisks (*p* < 0.05, *n* = 4–8). Scatter plots of the Spearman’s correlation test illustrate the age-related decline of the number (N) of CRF-FOSB immunoreactive (ir) cells upon ARS in the **(C)** CeA and **(D)** BNSTov. Boxed areas are depicted in higher magnification insets in the right top corner of the respective images. ic, internal capsule; st, stria terminalis. Scale bar 100 μm.

In order to test the acute stress sensitivity of CRF neurons of the extended amygdala, the number of FOS- and FOSB-containing CRF cells was quantified in the CeA and BNSTov. In these areas, the FOS ir was negligible in CRF cells even upon ARS exposure (see green panels in Figure 2), therefore, we do not present these data in details here. In contrast to the low FOS signal, considerable FOSB ir was detected in CRF cells of the CeA and BNSTov as detailed below.

In the CeA, ANOVA (Table 1) revealed the main effects stress (*F*_(__1__,__78__)_ = 6.04; *p* < 0.05) and age (*F*_(__7__,__78__)_ = 3.45; *p* < 0.01) on CRF-FOSB co-existence significant (Figure 2A). Although the effect was weak, the CRF-FOS cell count declined with age when 2M and 24M ARS rats were compared (*p* < 0.05). This was verified by a weak correlation (ρ = −0.42; *p* < 0.05, Figure 2C) also. The *post hoc* comparisons between control and ARS groups at various ages did not reveal a significant rise in FOSB cell counts in response to stress exposure. The CRF-FOSB cell count in ARS rats showed a strong positive correlation with BNSTov-CRF-FOSB cell count (ρ = 0.86; *p* < 10^–6^).

In the BNSTov, the number of CRF and FOSB co-localizing neurons was influenced both by acute stress (*F*_(__1__,__78__)_ = 4.13; *p* < 0.05) and age (*F*_7__,__78_ = 5.32; *p* < 10^–4^; Table 1). Tukey’s *post hoc* test showed a significantly lower cell count in 24M ARS compared both to 1M (*p* < 0.05) and 1.5M ARS animals (*p* < 0.01). However, the main effect of stress was significant; the *post hoc* tests did not confirm the ARS-induced elevation of FOSB (Figure 2B). Spearman’s test (Table 2) proved a negative correlation in ARS animals between CRF-FOSB cell number and age (ρ = −0.64; *p* < 0.00005, Figure 2D).

#### The CORT Response Upon Acute Restraint Stress Was Affected by Age

**FIGURE 3:**
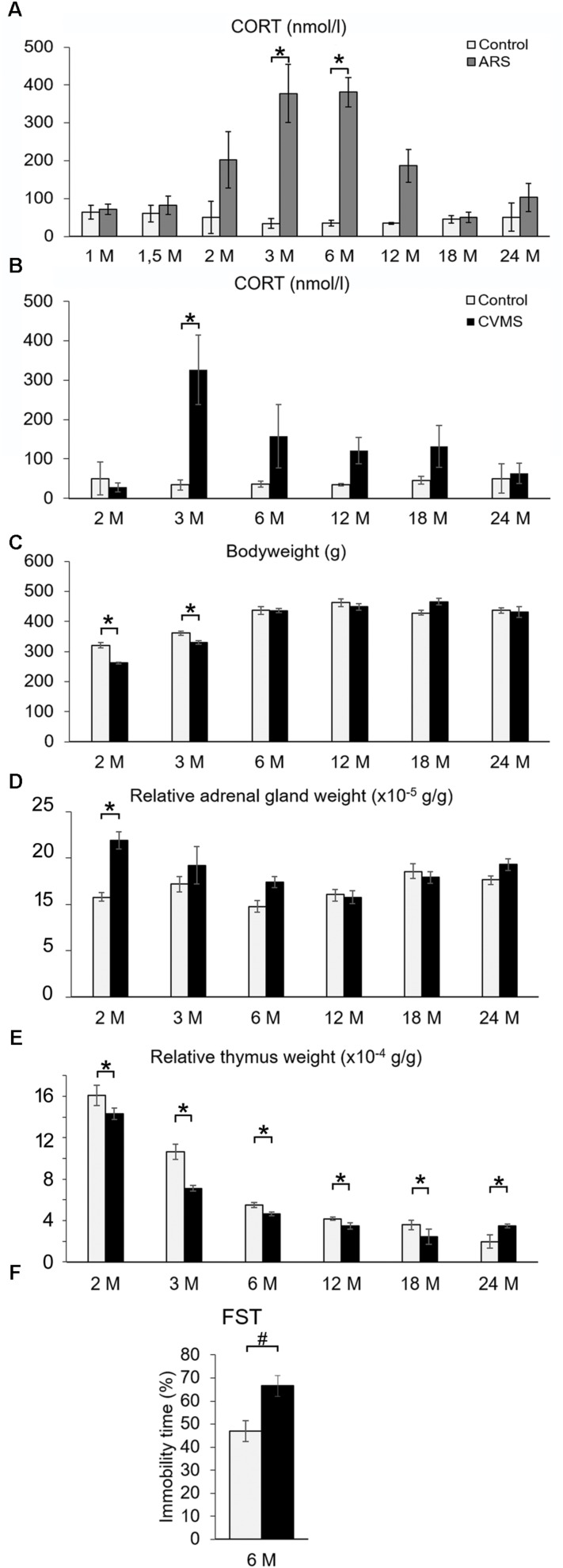
The efficacy of acute restraint stress (ARS) and chronic variable mild stress (CVMS) model. Panel **(A)** demonstrates corticosterone (CORT) levels upon ARS exposure (gray bars) vs. controls (open bars). Histogram **(B)** shows the effect of aging on CORT levels in CVMS exposed rats. Bodyweight **(C)** expressed in grams (g), relative adrenal weight **(D)**, relative thymus weight **(E)** changes as well as immobility time in forced swim test (FST) **(F)** support the effect of CVMS. Significant differences according to the Tukey’s *post hoc* test were marked by asterisks in panels **(A–E)** (*p* < 0.05, *n* = 4–8). The result of the FST in 6 months old (6M) rats **(F)** was assessed by Student’s *t*-test (#, *p* < 0.01, *n* = 12). Open bars represent control groups; black columns show data of CVMS exposed rats in histograms **(B–F)**.

In order to test if our ARS exposure activated the HPA axis, the plasma CORT content was determined. Both ARS exposure (*F*_(__1__,__78__)_ = 3.54; *p* < 0.01) and age (*F*_(__7__,__78__)_ = 30.73; *p* < 10^–5^) as well as their interaction (*F*_(__7__,__78__)_ = 4.55; *p* < 0.001) significantly influenced the CORT level (Table 3). 1M, 1.5M, 18M, and 24M rats did not show remarkable CORT elevation upon ARS exposure, while 3M and 6M animals (*p* < 0.0001) reacted with a significant CORT rise. In the case of 2M and 12M rats, the CORT level showed some increase upon ARS, but the elevation did not reach the statistically significant value according to the *post hoc* test (Figure 3A).

**TABLE 3:** Summary of statistical results obtained by two-way analysis of variance (ANOVA) to support the validity of our acute restraint stress (ARS) and chronic variable mild stress (CVMS) protocol.

	**Groups**	**Two-way ANOVA**					
		**Main effects**				**Interaction**	
		**Age**		**Stress**		**Age × stress**	
		***F*^∗^**	***p***	***F*^∗^**	***p***	***F*^∗^**	***p***
CORT level	Control vs. ARS	3.54	0.003	30.73	<10^–5^	4.55	<0.001
	Control vs. CMVS	3.02	0.018	13.96	<0.01	1.74	0.14
Body weight	Control vs. ARS	165.86	<10^–6^	1.14	0.29	3.07	<0.01
	Control vs. CMVS	60.54	<10^–6^	5.86	<0.05	10.96	<10^–6^
Relative adrenal weight	Control vs. ARS	57.77	<10^–6^	0.77	0.38	4.73	<0.001
	Control vs. CMVS	5.85	<5 ^∗^ 10^–4^	19.66	<5 ^∗^ 10^–4^	1.41	0.23
Relative thymus weight	Control vs. ARS	154.56	<10^–6^	0.23	0.63	2.04	0.06
	Control vs. CMVS	175.73	<10^–6^	0.28	0.60	4.00	<0.005

*^∗^The degrees of freedom for the comparison between control and ARS were *F*_(__1,78__)_ for stress. Moreover, *F*_(__7,78__)_ applied for or age and for age × stress interaction. In the control vs. CVMS comparison, the following degree of freedom values applied: *F*_(__1,63__)_ for stress and *F*_(__5,63__)_ for age and age × stress interaction, respectively. CORT, corticosterone.*

### Age Dependent Immediate Early Gene Product Content of CRF Neurons in the Chronic Variable Mild Stress Model

In order to test the hypothesis if the chronic stress reactivity of CRF neurons is also a function of age, the CVMS paradigm was tested also.

#### The Efficacy of Our Chronic Variable Mild Stress Protocol

To provide evidence that our CVMS was reliable, depression-like behavior in FST, plasma CORT level (Figure 3B), bodyweight (Figure 3C), and relative adrenal- (Figure 3D) and thymus weight data (Figure 3E) were assessed. The FST of CVMS-exposed 6M rats revealed that the stressor increased the animal’s depression-like behavior. The immobility time of control rats (46.97 ± 4.49%) increased to 66.55 ± 4.52% in CVMS exposed animals (Figure 3F; Student’s *t*-test: *p* < 0.01) supporting the validity of our CVMS protocol. CORT (Figure 3B and Table 3) was affected both by CVMS (*F*_(__1__,__63__)_ = 3.02; *p* < 0.02) exposure and by age (*F*_(__5__,__63__)_ = 13.96; *p* < 0.001) without an interaction (*F*_(__5__,__63__)_ = 1.74; *p* = 0.14). *Post hoc* comparisons revealed that the CVMS exposure caused a significant elevation in 3M rats. In older CVMS exposed animals, the CORT level decreased with age (Figure 3B).

Both CVMS (ANOVA: *F*_(__1__,__63__)_ = 5.86; *p* < 0.05) and age (*F*_(__5__,__63__)_ = 60.54; *p* < 10^–6^) as well as their strong interaction affected the animals’ total bodyweight (*F*_(__5__,__63__)_ = 10.96; *p* < 10^–6^). The CVMS-related bodyweight decline was significant when control and CVMS rats were exposed in the 2M and 3M groups (Tukey’s *post hoc* test, *p* < 0.05).

The main effect of both stress (*F*_(__1__,__63__)_ = 19.66; *p* < 10^–4^) and age (*F*_(__5__,__63__)_ = 5.86; *p* < 0.0005) influenced the relative adrenal weight (Figure 3D) without their interaction. Tukey’s *post hoc* test revealed the rise of relative adrenal weight upon CVMS in 2M rats significant (*p* < 0.05).

Analysis of variance confirmed that the thymus weight/bodyweights ratio was affected by age (*F*_(__5__,__63__)_ = 175.73; *p* < 10^–6^). The CMVS *per se* did not affect relative thymus weight, however, there was a marked effect of stress and age interaction (*F*_(__5__,__63__)_ = 4.00; *p* < 0.005). 2M control and CMVS animals showed significantly higher thymus weight/bodyweight ratio than all other groups (Tukey’s *post hoc* test, *p* < 0.0005), except 3M controls (Figure 3E).

#### CRF-FOSB Neurons Upon Chronic Variable Mild Stress

Six age groups of rats were subjected to CVMS vs. age-matched controls to study if there is an age-dependent change in the stress reactivity of CRF neurons. The comparison was performed in the same triple labeling for CRF, FOS, and FOSB. Our CVMS exposure did not induce FOS ir in the CRF neurons in any of the studied brain regions. As the FOS signal was negligible, we do not present these results in detail. In contrast, we found that CVMS affected the FOSB immunosignal in CRF cells. As animals were perfused 24 h after the last stress exposure, these FOSB data, in contrast to those in the above described ARS study, represent the ΔFOSB splice variant of the FOSB protein reflecting long-term changes in the cellular adaptation at transcriptional level (Kovács, 1998; Nestler, 2008).

**FIGURE 4:**
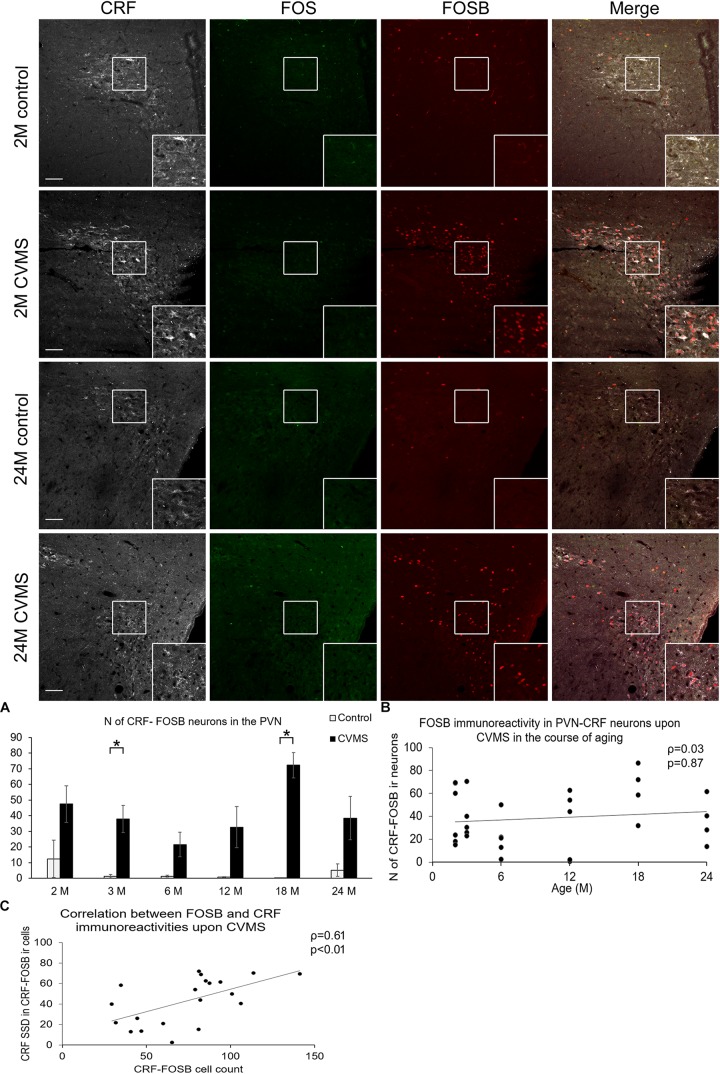
FOS and FOSB immunorectivities in the paraventricular nucleus of the hypothalamus (PVN). Representative images depicting the PVN area of 1-month-old (M) and 24M control and chronic variable mild stress (CVMS) exposed rats. Note that CRF (white) immunoreactive (ir) cells do not contain FOS (green). In contrast, they show FOSB (red) immunoreactivity upon CVMS as also shown by the merged image on the right. FOSB immunoreactivity does not decline with age as represented by images of 24M rats, and by histogram **(A)** that shows the number (N) of CRF and FOSB co-localizing cells in control (open bars) and CVMS exposed (dark columns) rats in six age groups. Significant differences according to the Tukey’s *post hoc* tests were highlighted by asterisks (*p* < 0.05, *n* = 4–8). Scatter plot **(B)** of the Spearman’s correlation test illustrates that there is no age-related decline of the N of CRF-FOSB labeled cells in CVMS. Graph **(C)** illustrates the positive correlation between FOSB immunoreactivity and the CRF content of the FOSB ir PVN neurons. Boxed areas are depicted in higher magnification insets in the right bottom corner of the respective images. 3rd, third ventricle. Scale bar 100 μm.

The number of cells which contained both CRF and FOSB was strongly affected by CVMS exposure in the PVN (ANOVA: *F*_(__1__,__63__)_ = 64.19; *p* < 10^–6^). In contrast, age did not influence the FOSB reactivity of CRF neurons (Figures 4A,B and Table 1). Tukey’s *post hoc* test confirmed the significant rise of CRF-FOSB cell counts upon CVMS in 3M and 18M groups (*p* < 0.0005) although a similar trend was observable in all age groups without statistical significance. In order to test if the FOSB affected the CRF content of the cells, the SSD of CRF ir was determined. We found that the CRF content of the cells correlated positively with the FOSB ir (ρ = 0.61; *p* < 0.01, Figure 4C). Contrary, the CRF content of cells which were negative for FOSB did not correlate (ρ = 0.24; *p* = 0.28) with the magnitude of FOSB signal.

#### FOSB Immunoreactivity in the CRF Neurons of the Central Amygdala Upon Chronic Variable Mild Stress

**FIGURE 5:**
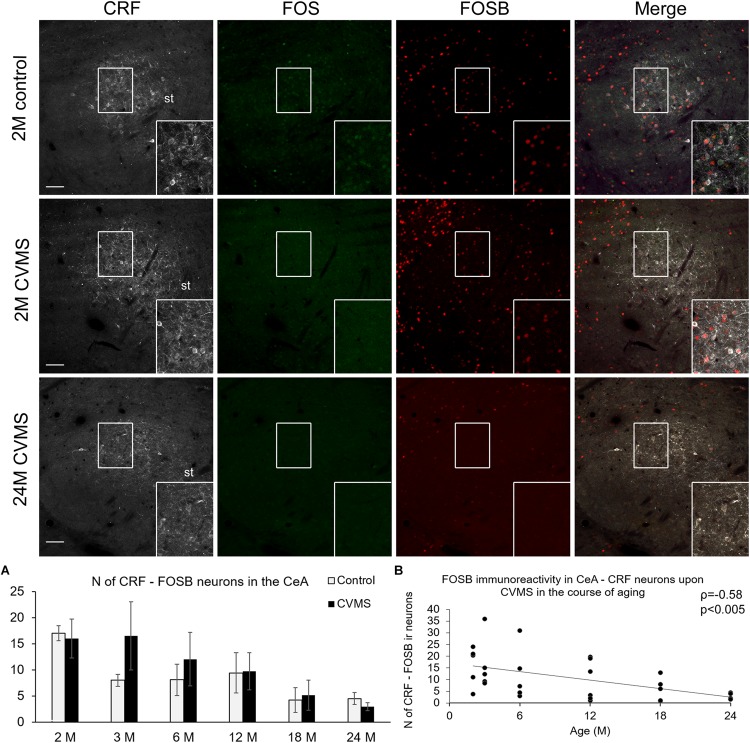
FOS and FOSB immunoreactivities in the central nucleus of amygdala (CeA). Representative images depicting the CeA area of 2-months-old (2M) control and chronic variable mild stress (CVMS) exposed rats compared with 24M rats upon CVMS. Note that CRF (white) cells are not immunoreactive (ir) for FOS (green). In contrast, in young rats, CeA-CRF cells show FOSB (red) immunoreactivity both in control rats and in animals upon CVMS exposure as also shown by the merged image on the right. The FOSB immunoreactivity declined with age as represented by images of 24M rats, and by histogram **(A)** that shows the number (N) of CRF and FOSB co-expressing cells in control (open bars) and CVMS exposed (dark columns) rats in six age groups. Scatter plot **(B)** of the Spearman’s correlation test illustrates the age-related decline of the N of CRF-FOSB labeled cells in CVMS. Boxed areas are depicted in higher magnification insets in the right bottom corner of the respective images. st, stria terminalis. Scale bar 100 μm.

The number of CeA cells co-localizing CRF and FOSB was affected by age (ANOVA: *F*_(__5__,__63__)_ = 3.93; *p* < 0.005) only (Table 1 and Figure 5A). The age-related decline of FOSB ir was confirmed by the correlation test also (ρ = −0.58; *p* < 0.005, Figure 5B and Table 4). We found a correlation between CeA-CRF-FOSB cell counts and relative thymus weight of the animals (ρ = 0.56; *p* < 0.005).

**TABLE 4:** Summary of statistical results by Spearman’s rank correlation tests in chronic variable mild stress exposed rats.

**Area**	**Labeling**	**Comparison with**	**ρ**	***p***
PVN	CRF-FOSB	Age	0.03	0.87
		Bodyweight	−0.02	0.92
		Relative adrenal weight	−0.02	0.92
		Relative thymus weight	−0.04	0.83
		CeA CRF-FOSB	0.04	0.82
		BNSTov CRF-FOSB	0.06	0.76
		CRF SSD in FOSB cells	0.61	<0.01
		CRF SSD in FOSB negative cells	0.24	0.28
BNSTov	CRF-FOSB	Age	−0.57	<0.01
		Bodyweight	−0.62	<0.001
		Relative adrenal weight	0.32	0.11
		Relative thymus weight	0.60	<0.01
		PVN CRF-FOSB	0.06	0.76
		CeA CRF-FOSB	0.82	<0.0001
CeA	CRF-FOSB	Age	−0.58	<0.01
		Bodyweight	−0.40	<0.05
		Relative adrenal weight	0.27	0.18
		Relative thymus weight	0.56	<0.01
		PVN CRF-FOSB	0.04	0.82
		BNSTov CRF-FOSB	0.82	<0.0001

*The number of FOSB-containing corticotropin-releasing factor (CRF) immunoreactive neurons of the paraventricular nucleus of the hypothalamus (PVN), the oval division of the bed nucleus of the stria terminalis (BNSTov) and central nucleus of the amygdala (CeA) was correlated with data depicted in the third column in CVMS exposed rats. SSD, specific signal density.*

#### FOSB Content of CRF Cells in the Oval Division of the Bed Nucleus of the Stria Terminalis Upon Chronic Variable Mild Stress

**FIGURE 6:**
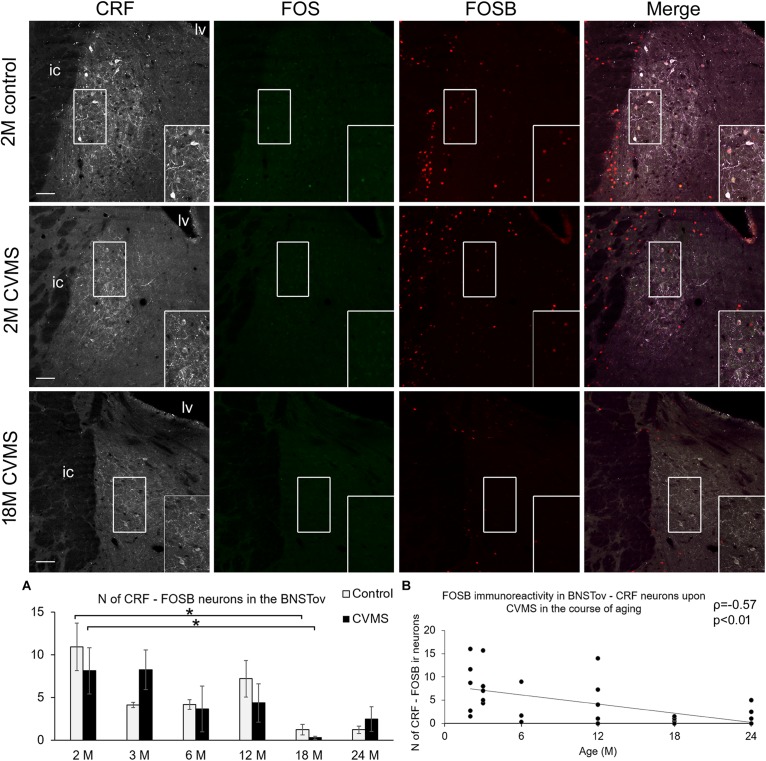
FOS and FOSB immunoreactivity in the bed nucleus of the stria terminalis (BNSTov). Representative images, depicting the BNSTov area of 2-months-old (2M) control and chronic variable mild stress (CVMS) exposed rats compared with 18M rats upon CVMS. Note that CRF (white) immunoreactive cells contain FOS (green) only occasionally. In contrast, in young rats, BNSTov-CRF cells show FOSB (red) immunoreactivity both in control rats and in animals upon CVMS exposure as also shown by the merged image on the right. The FOSB immunoreactivity declines with age as represented by images of 18M rats, and by histogram **(A)** that shows the number (N) of CRF and FOSB co-localizing cells in control (open bars) and CVMS exposed (dark columns) rats in six age groups. Significant differences according to the Tukey’s *post hoc* tests were highlighted by asterisks (*p* < 0.05, *n* = 4–8). Scatter plot **(B)** of the Spearman’s correlation test illustrates the age-related decline of the N of CRF-FOSB-labeled cells in CVMS. Boxed areas are depicted in higher magnification insets in the right bottom corner of the respective images. lv, lateral ventricle; ic, internal capsule. Scale bar 100 μm.

The assessment of CRF-FOSB ir in the BSNTov revealed that similar to the CeA, the number of CRF-FOSB double positive neurons was affected by age (*F*_(__5__,__63__)_ = 5.37; *p* < 0.001; Table 1). Tukey’s *post hoc* test found that the magnitude of CRF-FOSB cell count decreased with age (Figure 6A). The latter statement was supported by the Spearman’s correlation analysis also (ρ = −0.57; *p* < 0.005, Figure 6B). CeA and BNSTov CRF-FOSB cell counts correlated with each other (ρ = 0.81; *p* < 0.00005) and the latter with the relative thymus weight (ρ = 0.60; *p* < 0.005) also.

## Discussion

In this study we aimed at investigating the age dependency of FOS and FOSB response in acute and chronic stress models in CRF neurons of the PVN, CeA, and BNSTov in the rat. We focused on the possible age-dependent stress recruitment of CRF neurons. Our results support the hypothesis that the stress reactivity of CRF cells assessed by FOS and FOSB quantitation is a matter of age both upon acute and chronic stress. Results obtained in acute and chronic models will be discussed below.

### Validity of the Acute Stress Model

Besides other stress paradigms (Gaszner et al., 2004, 2009; Rouwette et al., 2011), ARS exposure in rats (Romeo et al., 2006; Meyza et al., 2007; Gaszner et al., 2009; Sterrenburg et al., 2012; Kovács et al., 2018) induces FOS ir in multiple brain areas. In line with earlier studies, here we found similar FOS activation proving prompt cell activation.

Corticosterone measurements also confirmed the efficacy of our ARS protocol. It has to be stated that in 1M and 1.5M rats ARS did not cause CORT rise that is in line with earlier studies using novel environment stress in Wistar rats (Koenig et al., 2018). The phenomenon that old Wistar rats do not show CORT level elevation in response to acute stress exposure is also known (Heine et al., 2004) and fits well with our present findings. The aging-related change of HPA axis stress responsivity seems to be strain dependent: in contrast to Wistar rats used in this study, Sprague-Dawley rats (Montaron et al., 2006) show increased CORT response in old age, in addition F344/Brown-Norway hybrid rats were shown to keep their stress reactivity till old age (Kasckow et al., 2005).

### Neural Activation of CRF Cells in the Paraventricular Nucleus Upon Acute Restraint Stress

The HPA axis, driven by CRF neurons of the parvocellular part of PVN, is affected by age (Romeo et al., 2006; Bao and Swaab, 2007; Aguilera, 2011). It is well documented that the number of CRF-FOS co-localizing cells in acute stress rises in the PVN (Crane et al., 2005; Romeo et al., 2006; Rotllant et al., 2007). The increase of FOS signal in PVN-CRF cells is lower in young (77-day-old) adults than in 28-days-old pre-pubertal animals (Romeo et al., 2006). In line with this, in the current study we saw that the ARS-induced PVN CRF-FOS co-existence decreased with the course of aging. To the best of our knowledge this is the first study to show that this trend continues till 24 months of age. In line with findings of our previous study using single FOS labeling (Kovács et al., 2018); in this work we add that the age-related decrease of FOS is characteristic for the CRF cells in the PVN.

We found that the magnitude of the CRF SSD of active PVN neurons correlated with their IEG content, contrary to the CRF SSD in IEG negative cells. This suggests that the decreasing FOS and FOSB in old age may contribute to the reduced CRF content of the PVN. This idea is supported by the fact that the transcription of the *Crf* gene is affected by AP1 (Malkoski and Dorin, 1999).

Although the FOS ir peaks earlier than FOSB (Kovács, 1998; Nestler, 2008), we did not see remarkable difference between FOS and FOSB cell counts in the acute model as their correlation was strong. This suggests that both markers have similar sensitivity to ARS that declines with age; therefore, the transcription of genes controlled by FOS and FOSB might also show lower expression in old age.

### Neural Activation of CRF Cells in the Central Amygdala Upon Acute Restraint Stress

In line with our (Kovács et al., 2018) and others (Butler et al., 2016) earlier studies, CeA shows increased FOS ir following acute restraint exposure. However, the FOS co-existence with CRF cells is also documented (Criado and Morales, 2000; Butler et al., 2016), others did not see the activation of CRF cells upon acute immobilization (Rotllant et al., 2007) and audiogenic stress (Helfferich and Palkovits, 2003). In line with the latter findings, we saw only very occasionally CRF-FOS co-localizations in the CeA (i.e., two to three cells per stressed animal corresponding to 2–4% of total CRF cell number). Taking this very low proportion of active cells into consideration, we assumed that the biological significance of these few active cells might be marginal. As the recruitment of FOS in CeA-CRF neurons upon acute stress was very low, and because CeA-CRF cells responded to the stress exposure in various models in earlier works (Rouwette et al., 2011; Sterrenburg et al., 2012), we decided to assess the FOSB protein content also. FOSB assessment revealed that CRF cells in the CeA exerted some basal FOSB signal that was affected by ARS, but the increase did not reach the level of significance in *post hoc* comparisons in none of the pairs of age groups. Interestingly, both basal and stress-evoked FOSB immunoreactivities declined with age to an almost undetectable level in senescence. When comparing the examined nuclei of the extended amygdala, the similar dynamics of CeA and BNSTov CRF cell activity may be explained by their direct connections (Dong et al., 2001) and similar GABAergic neurochemical character (Sparta et al., 2013; Partridge et al., 2016). On the other hand, the age-related dynamics of PVN-CRF neuronal activity showed a different pattern compared to the CRF cells in the CeA and BNSTov which may be explained by the glutamatergic phenotype of PVN-CRF cells (Hrabovszky et al., 2005).

### Neural Activation of CRF Cells in the Oval Division of the Bed Nucleus of the Stria Terminalis Upon Acute Restraint Stress

In line with the CeA, BNSTov neurons did not show remarkably increased FOS ir in CRF cells upon ARS. This is also in line with earlier works by other laboratories (Helfferich and Palkovits, 2003; Rotllant et al., 2007). In contrast, in our earlier study (Sterrenburg et al., 2012) we saw some FOS ir nuclei upon acute stress in the BNSTov; however, we did not perform co-localization assessment in that experiment. As the BNSTov besides CRF cells harbors stress responsive met-enkephalinergic neurons (Kozicz, 2002) future studies have to test if the weak FOS ir is characteristic for those met-enkephalinergic cells. Contrary to FOS, the number of CRF-FOSB co-localization was influenced by ARS, although the *post hoc* tests did not confirm a significant elevation in any age. Interestingly, the FOSB content of BNSTov neurons declined with age, and finally it disappeared in older age. The BNSTov CRF cells are mostly GABAergic inhibitory neurons projecting to the PVN (Sparta et al., 2013; Dabrowska et al., 2016; Romanov et al., 2017; Sunstrum and Inoue, 2018). The BNSTov CRF neurons also send fibers both to the dorsal raphe nucleus (Weissbourd et al., 2014; Dabrowska et al., 2016) and LC (Van Bockstaele et al., 1999). Future work has to unravel if the aging-related decline of BNSTov-CRF neuronal activity contributes to aging-related dynamics in the activity of the PVN and brainstem stress centers.

### The Validity of the Chronic Variable Mild Stress Model

In line with earlier studies in mice (Bali and Jaggi, 2015; Kormos et al., 2016; Farkas et al., 2017) and rats (Romeo et al., 2006; Sterrenburg et al., 2011), CVMS affected bodyweight gain that was supported by *post hoc* tests in 2M and 3M rats only. This might be explained by the fast growth of rats in this age that is blunted by catabolic effects of elevated glucocorticoid levels related to increased HPA axis activity. However, in older age, the effect of CVMS on bodyweight change was not significant, the relative thymus and adrenal weight data showed the effect of CVMS more reliably, except for the oldest group. We also found that the relative thymus weight correlated with the FOSB content of the examined extrahypothalamic CRF neurons. This suggests that the reduced FOSB content in aged CRF cells of the BNSTov and CeA might contribute to a higher activity of the HPA axis. Controversial literature data are available on the change of relative thymus weight in similar studies (Romeo et al., 2006; Carvalho-Netto et al., 2011; Herman et al., 2016; Wulsin et al., 2016). Here we show that besides several other factors (i.e., species, strain, type of stressor used), age, and consequent bodyweight differences (i.e., gain in young rats, loss in old age, Füredi et al., 2018) as well as thymus involution in old animals (Nacka-Aleksić et al., 2019) might interfere with the reliability of these indicators of stress efficacy. Based on these we propose that the body- and relative organ weight data as physical indicators of chronic stress efficacy are highly sensitive in young animals, but they lose their reliability in old age.

The increase of FOSB ir in CRF neurons confirmed the reliability of our CVMS model in all age groups that is in line with findings of other studies (Imaki et al., 1991; Herman et al., 1995; Kormos et al., 2016; Wulsin et al., 2016). Importantly, the FOSB ir positively correlated with the CRF SSD in the PVN. This is not surprising in the view of the fact that the promoter of the *Crf* gene contains multiple AP1 binding sites (Malkoski and Dorin, 1999). Our finding that the CRF content of FOSB negative PVN cells did not correlate with the magnitude of FOSB expression suggests that FOSB is indeed required for the increase of CRF content in PVN cells in chronic stress. Moreover, our CORT data further support the increased activity of the HPA axis, and the efficacy of the CVMS exposure in young adult rats.

In line with results of earlier studies (Bowman et al., 2006), we found that our old rats do not react with significant CORT elevation to CVMS exposure. In accord with our last study (Kovács et al., 2018), we did not see any strong correlation of CORT data and the neuronal activity in the examined regions (data not shown). This suggests that the histological evidence for IEG content of CRF cells in the examined nuclei does not mirror the magnitude of HPA axis activity. However, we did all efforts to avoid this, it has to be stated, that in this experimental setup one cannot rule out that to some extent acute stress effect related to the anesthetic injection could have biased the CORT values of rats in the CVMS model. This might have abolished correlations between CORT values and the magnitude of CRF neuronal activity.

Our FST supported that the CVMS exposure increased the depression-like phenotype. Although this further supports the validity of our model, an important limitation of our behavioral findings is that due to capacity limitations we cannot provide FST data in all age groups.

Collectively, these data support that CVMS exposure was effective in this experiment, but the sensitivity of physical and endocrinological parameters exerted age-related limitations. In this setup, we saw that the relative thymus weight was the most sensitive indicator of stress efficacy throughout the lifespan.

### The FOSB Neuronal Activity of CRF Cells in the Paraventricular Nucleus Following Chronic Variable Mild Stress Is Not Affected by Age

In line with previous studies performed in mice (Kormos et al., 2016) and rats (Sterrenburg et al., 2012) here we also saw increased PVN-FOSB signal upon CVMS (de Andrade et al., 2014). Sterrenburg et al. (2012) as well as Radley and Sawchenko (2015) showed that chronic variable stress increased both CRF and FOSB (ΔFosB) ir in the PVN. Kiss and Majercikova (2017) also proved the co-localization of these two antigens in the PVN. In contrast to the FOS (Kovács et al., 2018) and glucocorticoid receptor content (Mizoguchi et al., 2009) our current data suggest that the reactivity of the PVN as assessed by CRF-FOSB labeling is not a function of age, as CVMS exposed animals show high double-labeled cell count till old age. We showed here also that the FOSB ir cells contained more CRF, suggesting that the FOSB protein stimulates the expression of *Crf* that is in line with earlier findings (Kageyama et al., 2014). The comparison of age-related changes in *Crf* mRNA levels (Tenk et al., 2017) with the FOSB neuronal activity in CRF neurons (this study) revealed that 3M, 12M, 18M, and 24M CVMS animals displayed similar dynamics. Future molecular experiments have to determine how ΔFOSB interacts with the AP-1 binding sites of the C*rf* gene promoter (Malkoski and Dorin, 1999) in the course of aging.

### The Neuronal Activity of CRF Cells in the Central Amygdala and in the Oval Division of the Bed Nucleus of the Stria Terminalis Does Not Increase Upon Chronic Variable Mild Stress, but Decreases With Age

In the present study, we did not observe remarkable alteration of FOSB (ΔFOSB) content of CRF neurons neither in CeA nor in BNSTov upon CVMS exposure. This is in line with our (Sterrenburg et al., 2011) and others (Majercikova and Kiss, 2015) earlier works where the CRF and FOSB were assessed in young adult rats. The FOSB data indicate that some CRF neurons in the CeA and BNSTov were active in both control and CVMS animals. The number of these FOSB ir CeA and BNSTov CRF neurons strongly decreased with aging in both control and CVMS animals. This was in contrast with findings in the PVN, where we did not see remarkable basal FOSB activity in controls. This difference in the stress sensitivity of CRF neurons might be attributed to the different neurochemical character of these (Sparta et al., 2013; Partridge et al., 2016; Deussing and Chen, 2018). Indeed, it was shown that tonic inhibitory neurons do not display increase in *Fos* gene product content upon environmental stimuli (Reisch et al., 2007). The significance of aging-related decrease of CeA and BNSTov neuronal activity is to date unknown.

### FOS Proteins as Neuronal Activity Markers Show Different Age Dependent Dynamics

In the present study we also detected FOS in the ARS model at 2 h successfully, which is in line with earlier studies (Romeo et al., 2006; Meyza et al., 2007; Gaszner et al., 2009; Sterrenburg et al., 2012; Kovács et al., 2018). One of the main new findings of this study is that the age-related decline of FOS reactivity is characteristic for the CRF neurons in the PVN. FOSB protein was shown to increase in few hours after the stimulus but it exerts a longer half-life of 9.5 h (Kovács, 2008). Indeed, we successfully detected the FOSB ir that highly co-localized with FOS in the PVN-CRF cells. In this study, we demonstrated first also that the acute stress exposure-evoked PVN-CRF activation that recruits both FOS and FOSB proteins declines with age also. The ΔFOSB splice variant of FOSB is widely used to detect long-term changes in the neuronal activation. In our CVMS model we euthanized our rats 24 h after the last stress exposure in order to avoid the acute effect and consequent acute FOSB elevation that would be detected by our antibody also (Sterrenburg et al., 2011). This approach allowed us to show first time that the ΔFOSB isoform of FOSB does not show age-dependent decline in the PVN, in contrast to the acute activity markers (i.e., FOS and full-length FOSB).

### Limitations of Our Results

First, the visualization of FOS proteins provides information about the activity of the AP-1 complex (Kovács, 1998), but does not refer to the activity of other intracellular signaling pathways. Second, the technique does not provide adequate information about the exact cell function either, since AP-1 regulates numerous cellular functions at transcriptional level (Nestler, 2008). Third, in the ARS model we measured both FOS and FOSB immunosignal at 2 h. However, for the FOSB, this time point represents the initial phase of its rise (Kovács, 1998, 2008; Nestler, 2008). Therefore, one could expect that more CRF cells could have developed FOSB ir if we would have collected the brains in a later time point. Because of ethical reasons, technical considerations related to the large sample size and limitations of our animal facility we decided to euthanize the ARS rats 2 h after the start of the stress exposure. Fourth, we do not provide in all age groups behavioral data to support the depression-like phenotype upon CVMS exposure.

## Conclusion

To the best of our knowledge, this is the first study providing a systematic throughout-lifespan description of neuronal activity in the main hypothalamic and forebrain CRF systems in response to ARS and CVMS in the rat. The neuronal activity of the examined CRF cells was found to be a function of age and brain area. CVMS was found to cause an age-independent FOSB/ΔFOSB neuronal activity in PVN-CRF cells. Therefore, studies applying FOS and FOSB as activity markers should be planned with respect to the age and brain region-specific recruitment of these indicators. Further studies are in progress to describe the aging-related alteration of neuronal stress responsivity in other stress-recruited circuits. Getting an overview on the age-dependency of stress-reactive areas may help to understand why do stress-related brain diseases occur more frequently in adolescence and in old age. This knowledge might ultimately help to find new personalized, eventually age-adjusted strategies for prevention and management of stress-related mood-disorders.

## Data Availability Statement

The datasets generated for this study are available on request to the corresponding author.

## Ethics Statement

The animal study was reviewed and approved by the Ethics Committee on Animal Research of Pécs University.

## Author Contributions

LK performed the animal experiments, evaluated the results, performed the statistics, prepared the figures, and wrote the draft of the manuscript. NF and BU performed the immunolabeling, digital imaging, and morphometry. GB contributed to the work at the confocal microscope. VC performed and validated the corticosterone measurement. BG designed the experiments, collected the blood samples, performed the perfusion, supervised the tissue preparation, imaging, selected the images containing the areas of interest, helped with figure preparation, and supervised the manuscript.

## Conflict of Interest

The authors declare that the research was conducted in the absence of any commercial or financial relationships that could be construed as a potential conflict of interest.
