# Supplementary figures and images for: Corticotropin-Releasing Factor-Producing Cells in the Paraventricular Nucleus of the Hypothalamus and Extended Amygdala Show Age-Dependent FOS and FOSB/DeltaFOSB Immunoreactivity in Acute and Chronic Stress Models in the Rat
Source: Front Aging Neurosci. 2019 Oct 9;11:274. doi: 10.3389/fnagi.2019.00274 (PMC6794369; doi:10.3389/fnagi.2019.00274)

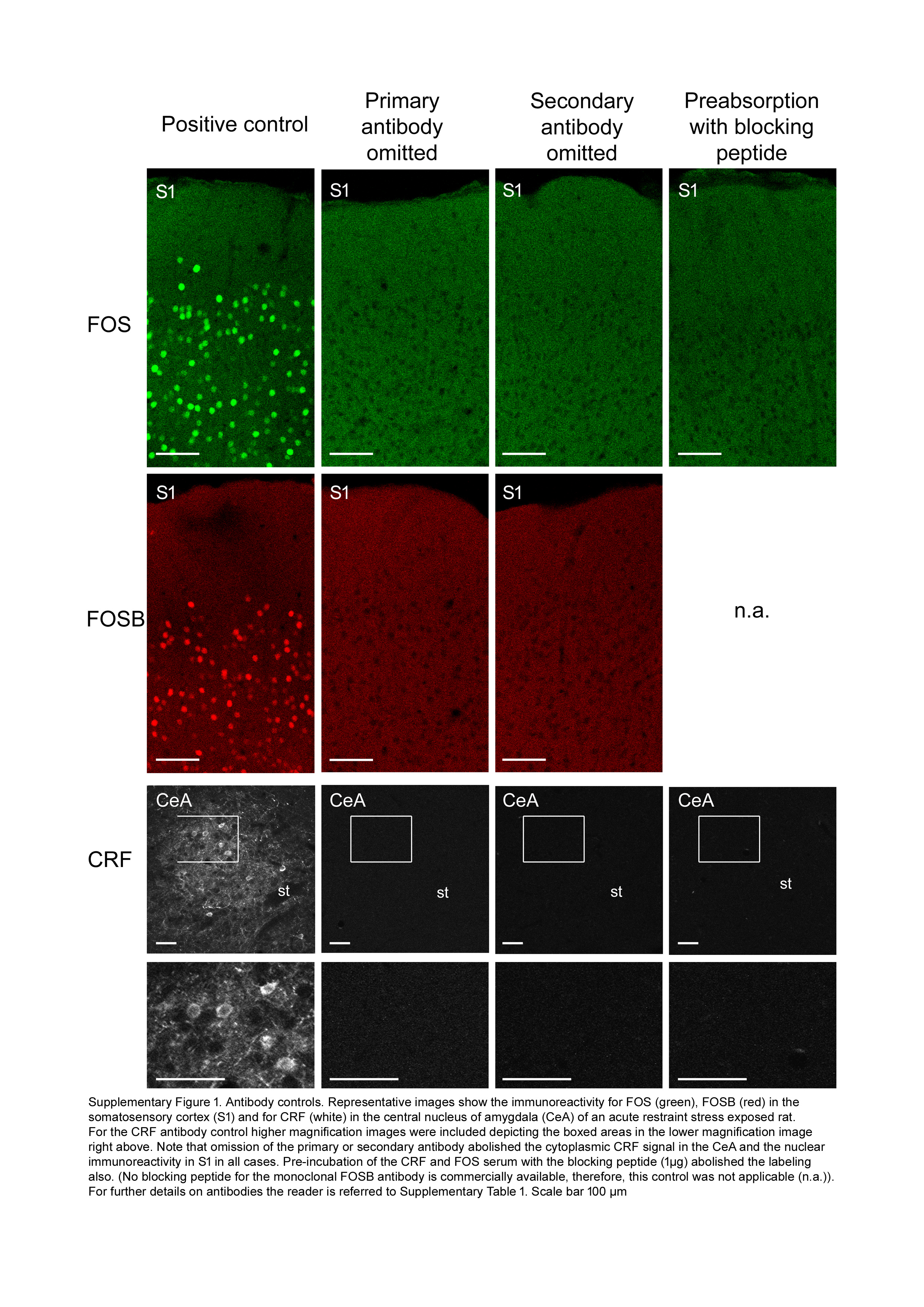

Supplement: Supplementary file 1 [file Image_1.jpg]
