# Supplementary material for: Corticotropin-Releasing Factor-Producing Cells in the Paraventricular Nucleus of the Hypothalamus and Extended Amygdala Show Age-Dependent FOS and FOSB/DeltaFOSB Immunoreactivity in Acute and Chronic Stress Models in the Rat
Source: Front Aging Neurosci. 2019 Oct 9;11:274. doi: 10.3389/fnagi.2019.00274 (PMC6794369; doi:10.3389/fnagi.2019.00274)
Supplement: Supplementary file 2 [file Table_1.docx]

| **Antibody name** | **Vendor** | **Cat#** | **Reference** | **RRID** | **Antigen** | **Dilution** |
| --- | --- | --- | --- | --- | --- | --- |
| Anti-c-Fos antibody  (host: guinea pig, polyclonal) | Synaptic Systems GmbH | 226004 | Octeau et al 2019 Cell Rep. 27:2249-2261  PMID:31116972 | AB_2619946 | Synthetic peptide corresponding to AA 2 to 17 from rat c-Fos (UniProt Id: P12841) | 1:1000 |
| Anti-Fos B antibody [83B1138] (host: mouse, monoclonal) | Abcam | ab11959 | Tuplin et al 2018. [Front Behav Neurosci](https://www.ncbi.nlm.nih.gov/pmc/articles/PMC6055009/). 12: 144  PMID: 30061817 | AB_298732 | synthetic peptide corresponding to human Fos B | 1:1500 |
| Anti-CRF antibody (host: rabbit polyclonal) | Peptide Biology Lab.; Salk Insitute | PBL# rC70 | Justice et al 2008 J Comp Neurol. 2008 511:479-96  PMID: 18853426 | AB_2314234 | full-length rat/human CRF(1-41) | 1:16000 |
| Alexa Fluor 488 AffiniPure donkey anti-guinea pig IgG (H+L) | Jackson Immunoresearch | 706-545-148 | Stratford et al 2014. PLoS One. 9(9):e107238.  PMID: 25192442 | AB_2340472 | - | 1:600 |
| Cy3 AffiniPure Donkey Anti-Mouse IgG (H+L) | Jackson Immunoresearch | 715-165-150 | Toossi et al 2016 9;3(3). ENEURO.0077-16.2016.  PMID: 27294196 | AB_2340813 | - | 1:500 |
| Biotin-SP (long spacer) AffiniPure donkey anti-rabbit IgG (H+L) | Jackson Immunoresearch | 711-065-152 | Li et al 2015  Endocrinology. 2015 156:2807-20.  PMID: 25978516 | AB_2340593 | - | 1:500 |

Supplementary Table 1. Detailed information on antibodies used in the study. (References with full bibliographic information are included into the reference list of the paper).

References for Suppl. Table 1.

Octeau JC, Gangwani MR, Allam SL, Tran D, Huang S, Hoang-Trong TM, Golshani P, Rumbell TH, Kozloski JR, Khakh BS. Transient, Consequential Increases in Extracellular Potassium Ions Accompany Channelrhodopsin2 Excitation. Cell Rep. 2019 May 21;27(8):2249-2261 doi: 10.1016/j.celrep.2019.04.078

[Tuplin](https://www.ncbi.nlm.nih.gov/pubmed/?term=Noye%20Tuplin%20EW%5BAuthor%5D&cauthor=true&cauthor_uid=30061817) EWN, [Lightfoot](https://www.ncbi.nlm.nih.gov/pubmed/?term=Lightfoot%20SH%5BAuthor%5D&cauthor=true&cauthor_uid=30061817) SHM, [Holahan](https://www.ncbi.nlm.nih.gov/pubmed/?term=Holahan%20MR%5BAuthor%5D&cauthor=true&cauthor_uid=30061817) MR (2018) Comparison of the time-dependent changes in immediate early gene labeling and spine density following abstinence from contingent or non-contingent chocolate pellet delivery [Front Behav Neurosci](https://www.ncbi.nlm.nih.gov/pmc/articles/PMC6055009/). 12: 144. https://doi: [10.3389/fnbeh.2018.00144](https://dx.doi.org/10.3389%2Ffnbeh.2018.00144)

Justice NJ, Yuan ZF, Sawchenko PE, Vale W. Type 1 corticotropin-releasing factor receptor expression reported in BAC transgenic mice: implications for reconciling ligand-receptor mismatch in the central corticotropin-releasing factor system. J Comp Neurol. 2008 Dec 1;511(4):479-96. doi: 10.1002/cne.21848.

Stratford JM, Thompson JA. Beta-galactosidase staining in the nucleus of the solitary tract of Fos-Tau-LacZ mice is unaffected by monosodium glutamate taste stimulation. PLoS One. 2014 Sep 5;9(9):e107238. doi: 10.1371/journal.pone.0107238

Toossi H, Del Cid-Pellitero E, Jones BE GABA Receptors on Orexin and Melanin-Concentrating Hormone Neurons Are Differentially Homeostatically Regulated Following Sleep Deprivation. eNeuro. 2016 Jun 9;3(3). pii: ENEURO.0077-16.2016. doi: 10.1523/ENEURO.0077-16.2016

Li AJ, Wang Q, Elsarelli MM, Brown RL, Ritter S. Hindbrain Catecholamine Neurons Activate Orexin Neurons During Systemic Glucoprivation in Male Rats. Endocrinology. 2015 Aug;156(8):2807-20. doi: 10.1210/en.2015-1138
